# Impact of diet on the gut mycobiome of Hong Kong Chinese infants

**DOI:** 10.1016/j.csbj.2025.02.006

**Published:** 2025-02-14

**Authors:** Jordan Yik Hei Fong, Kris Yuet Wan Lok, Man Lung Yeung, Wing Ho Li, Patrick Chiu Yat Woo, Jade Lee Lee Teng

**Affiliations:** a Faculty of Dentistry, The University of Hong Kong, Hong Kong, China; b Department of Microbiology, School of Clinical Medicine, Li Ka Shing Faculty of Medicine, The University of Hong Kong, Hong Kong, China; c Centre for Virology, Vaccinology and Therapeutics, Hong Kong Science and Technology Park, Hong Kong, China; d School of Nursing, the University of Hong Kong, Hong Kong, China; e Pandemic Research Alliance Unit at The University of Hong Kong, The University of Hong Kong, Hong Kong Special Administrative Region, China; f State Key Laboratory of Emerging Infectious Diseases, Li Ka Shing Faculty of Medicine, The University of Hong Kong, Hong Kong, China; g Department of Clinical Microbiology and Infection Control, The University of Hong Kong-Shenzhen Hospital, Shenzhen 518053, China; h Carol Yu Centre for Infection, Li Ka Shing Faculty of Medicine, The University of Hong Kong, Hong Kong, China; i Doctoral Program in Translational Medicine and Department of Life Sciences, National Chung Hsing University, Taichung 402, Taiwan; j The iEGG and Animal Biotechnology Research Center, National Chung Hsing University, Taichung 402, Taiwan

**Keywords:** Hong Kong Chinese infants, Diet, Gut, Mycobiome, Fungi, Breastmilk

## Abstract

Despite extensive research on the gut bacteriome during infancy and its correlation with various chronic diseases, the influence of diet on gut mycobiome development in infants remains unexplored. To address this significant research gap, we conducted a study on 70 healthy Hong Kong Chinese infants who were either directly breastfed, expressed milk-fed, or formula-fed. Our analysis revealed that formula-fed infants had higher fungal diversity and composition in their gut mycobiome compared to those in breastfed and expressed milk-fed infants. The formula-fed group exhibited higher fungal richness, with a median of 58.5, compared to a median of 34 in the breastfed group (*p* = 0.04) and 28.5 in the expressed milk-fed group (*p* = 0.02). However, the breastfed and expressed milk-fed groups showed no significant differences. In terms of fungal compositions, formula-fed infants had a significant increase in the relative abundance of *Pochonia* (0 % in breastmilk vs 0.5 % in formula milk, false discovery rate (FDR)-corrected *p* = 0.05), *Saccharomyces* (0.95 % in breastmilk vs 2.7 % in formula milk, FDR corrected *p* = 0.03), and *Tetrapisispora* (0.6 % in breastmilk vs 3.0 % in formula milk, FDR corrected *p* = 0.002), whereas direct breastfed infants exhibited an increased abundance of *Malassezia* (breastmilk vs. formula milk = 1.4 % vs. 0.4 %, FDR-corrected *p* = 0.01). Overall, our results indicate that the composition of breastmilk and formula milk can have varying impacts on the gut mycobiome of infants, providing new insights into the diet-gut mycobiome dynamic in early life. Given the small sample size of the expressed milk group, the findings of this group should be considered preliminary or exploratory. Further studies are thus necessary to explore the potential health implications of these findings.

## Highlights

• The influence of diet on the infant gut mycobiome remains unexplored.
• Mycobiome analysis showed higher diversity in formula-fed infants.
• Overall, breastmilk and formula impact the gut mycobiome of infants differently.

## Graphical Abstract

**Figure:**
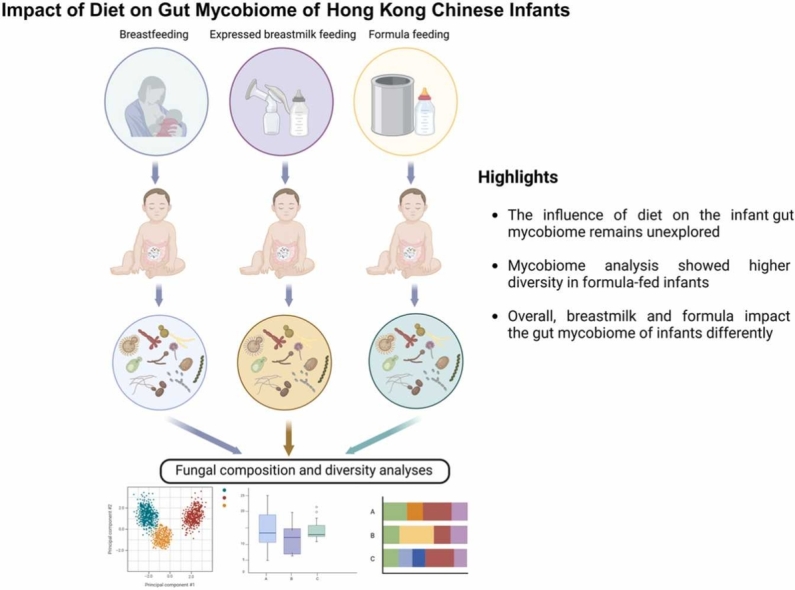

## 1 Introduction

The gut microbiome, a complex ecosystem of microorganisms residing in the human digestive tract, plays a pivotal role in shaping human physiology, immunological functions, and metabolic activity [1], [2], [3]. Given its critical role, this microbial community and its potential impact on human health has been extensively investigated [1], [4], [5], [6], [7], [8]. Recent findings have revealed that an abnormal microbiome, termed dysbiosis, is strongly associated with the incidence of chronic diseases, such as inflammatory bowel disease, obesity, asthma, diabetes, and colorectal cancer, in adults [9], [10], [11], [12], [13].

Beyond its well-studied impact on adult physiology, the gut microbiome plays a crucial role in infant development, a period during which its structure remains highly vulnerable. Dysbiosis during this period has been linked with the colonization of opportunistic pathogens and the development of chronic diseases such as obesity and immune disorders, leading to potentially long-term consequences [14], [15], [16], [17]. Several factors have been identified as potential influencers of the infant gut microbiota, including diet, delivery method, antibiotic usage, environmental conditions, and geographic location [18], [19], [20], [21], [22], [23], [24]. Among these, diet, particularly maternal breastmilk, has the most significant impact on the growth and composition of the gut microbiome, as it provides essential micronutrients to support its development and provide physiological protective functions [24], [25], [26], [27].

Extensive research has explored the influence of diet, particularly breastmilk feeding, on the infant gut bacterial microbiome (i.e., bacteriome). However, limited data exist about its impact on the broader microbial community, including fungi and viruses [28], [29]. Among these, the gut fungal microbiome, also known as the mycobiome, has recently gained recognition for its potential role in human health and disease [30], [31], [32], [33]. The gut mycobiome, typically present in low abundance, accounts for less than 0.1 % of the total gut microbiome. In infants, *Saccharomyces*, *Malassezia*, and *Candida* are predominant fungal genera commonly found in the gut mycobiome. Their colonization is influenced by factors such as delivery mode and maternal fungal transmission during birth [31], [33]. Further, breastmilk has also been reported to carry various fungal species. However, transmission of the breastmilk mycobiome and its impact on infants remain to be investigated [34]. Notably, dysbiosis of the gut mycobiome in early life has been associated with allergic diseases, inflammatory bowel disease, and obesity [30], [31], [32], [33], [35].

Understanding how diet influences the gut mycobiome is a critical research area, particularly when the gut microbiome is rapidly developing. While the relationship between diet and the gut bacteriome is well-established, our understanding of how diet shapes the gut mycobiome remains limited. This research gap is critical considering the recognized influence of maternal breastmilk on the development of the infant gut microbiome during early life [24], [36], [37]. However, the impact of diet, whether through breastfeeding, expressed breastmilk, or formula feeding on the infant gut mycobiome remains unexplored, despite the global prevalence of diverse infant feeding practices [38], [39], [40], [41]. In Hong Kong, expressing breastmilk has gained significant popularity, with over 80 % of mothers engaging in this practice [42]. Similarly, a recent study in Hong Kong public hospitals shows that approximately 60 % infants receive supplementary infant formula before leaving the hospital, indicating that use of varied feeding methods remains common in Hong Kong [43]. However, the effects of different feeding practices on the gut mycobiome of Hong Kong infants have not been investigated.

Investigating the gut mycobiome, however, presents unique challenges owing to the limitations of current methodologies. Most studies rely on the amplicon sequencing of ribosomal DNA, such as sequencing of the internal transcribed spacer (ITS) region for fungal profiling [44], [45], [46]. Although this approach is widely used, amplicon sequence-based estimation may be influenced by primer bias and size selection [47], [48]. To overcome these limitations, shotgun metagenomic sequencing may offer a more suitable strategy [48], [49].

Therefore, this study aimed to analyze the gut mycobiome of infants in Hong Kong and to explore the impact of diet feeding modes, including breastfeeding, expressed milk feeding, and formula milk feeding, on fungal diversity and composition. In this study, by employing shotgun metagenomic sequencing, we aim to characterize the mycobiome composition among healthy Chinese infants and gain insights into the early infant mycobiome in different dietary groups. This would contribute to the limited knowledge on the gut mycobiome and its relationship with infant diets, specifically in the context of Hong Kong infants.

## 2 Materials and methods

### 2.1 Study participants

We included 70 Chinese infants, representing a subset of a recent infant feeding study [50]. Women who were local Hong Kong residents, 18 years of age or older, and had singleton pregnancies were recruited from three postnatal wards between August 2018 and December 2019. Mothers who had a history of serious medical or obstetrical complications were excluded from the study. Participants interested in the study were asked about their infant feeding diet and medication use from birth to screen for mother-infant eligibility. Infants at the age of six weeks, categorized into three feeding groups (breastfeeding, expressed breastmilk feeding, and formula feeding), were recruited for the study [50]. To be eligible for inclusion in the study, infants had to be healthy with at least 37 weeks of gestation. In total, 30 samples from breastfed infants, 10 from expressed milk-fed infants, and 30 from formula-fed infants were collected. Basic demographics, maternal and infant data, including infant sex, birth weight, gestational age, birth mode (caesarean section or vaginal delivery), parity, medication, and basic infant health and feeding data) were collected from medical records or through self-reporting by the participants. Infants who were fed both formula and breast milk, or cases in which the infant or mother had received medications known to impact microbiota, were excluded from the study.

### 2.2 Collection of infant fecal samples, DNA extraction, and high-throughput sequencing

Fecal samples were collected from infants at six weeks of age as part of a scheduled home visit. For sample collection, mothers were instructed to use a non-absorbent liner in their infant’s diaper on the day before the home visit and to change the liner with the diaper until stool was obtained. The liner-containing diaper was placed in a collection bag, sealed, and stored in a refrigerator until a trained research assistant collected the sample using sterile screw cap containers and a scoop. Once the sample was collected, it was immediately transported to the laboratory on ice and frozen at −80 °C prior to further processing.

DNA extraction was performed using the QIAamp PowerFecal DNA kit (QIAGEN); 200 mg of each stool sample was aliquoted into provided bead tubes according to the manufacturer’s protocol. The samples were then vortexed at maximum speed (30 Hz) for 10 minutes using the QIAGEN TissueLyser II, followed by DNA extraction. The extracted genomic DNA was fragmented using the Diagenode Bioruptor Pico system, resulting in a peak size of approximately 300 bp. The fragmented DNAs were ligated with an IDT dual-indexed UMI adaptor system. The adapter-ligated library, with a size range of 300–750 bp, was selected using the dual-SPRI method. All libraries were prepared according to the protocols of the KAPA HyperPrep Kit (KR0961). The enriched libraries were subjected to quality control and validation using the Agilent Bioanalyzer, Qubit, and qPCR. Illumina NovaSeq 6000 was used for Pair-End 151 bp sequencing.

### 2.3 Fecal mycobiome analysis

The fecal mycobiome was analyzed relative to the total fungal abundance to specifically characterize the mycobiome dynamics, independently of bacterial abundance, which was extensively analyzed in our previous study [50]. The raw reads generated using Illumina NovaSeq 6000 were processed using FASTP version 0.20.2 to filter out low-quality reads [51]. The filtered reads were then used to profile the fungal community using Kraken2 version 2.1.2 and Bracken version 2.5 [52], [53], with the Kraken fungal RefSeq database (k2_pluspf_20231009) obtained from https://benlangmead.github.io/aws-indexes/k2 [52], [54]. Species profiles were analyzed using the *phyloseq* R package, which compiled the features table, taxonomy data, and subject metadata. The taxonomic strata were further analyzed using *phyloseq* and visualized using *ggplot2* packages [55], [56]. To assess the alpha diversity of the gut mycobiome, observed features and Shannon diversity, which measure species richness and diversity, were calculated using functions from the *vegan* package [57]. Beta diversity indices were calculated based on feature profiles using Bray-Curtis dissimilarity and were visualized using principal coordinate analysis (PCoA) in the *vegan* and *ecodist* packages [57], [58].

### 2.4 Statistical analysis

To compare microbiota measures, including alpha diversities and fungal relative abundances, between feeding status groups, we employed non-parametric Kruskal-Wallis tests, Mann-Whitney *U* test, and Dunn's tests with false discovery rate (FDR) correction using *rstatix* package [59]. Fungal beta diversities were assessed using permutational analysis of variance (PERMANOVA) on Bray-Curtis distance matrices, with 9999 permutations, implemented in the *vegan* package [57]. Pairwise PERMANOVA between groups was calculated using the *EcolUtils* R package [60]. We adjusted for potential confounders, including gender, mode of delivery, and age of infants, using multiple linear regression, multivariate PERMANOVA, and multivariable logistic regression models with the *stats* and *vegan* packages [57]. The presence of statistically significant fungal features was evaluated using the pairwise chi-square test, odds ratio (OR) and the Wald chi-square test, based on prevalence data and a multivariable logistic regression model. All data analysis and visualizations were performed using R version 4.3.2.

## 3 Results

### 3.1 Population characteristics

In total, 70 infants were recruited for analysis in this study, including 30 infants who were directly breastfed (13 male, 17 female), ten infants who were fed expressed milk (5 male, 5 female), and 30 infants who were formula-fed (14 males, 16 females). Statistically significant differences were observed between different groups in relation to the mode of delivery (Table 1). The formula-fed group had a higher proportion of infants born via C-section compared to that in the breastfed group (*p* = 0.03). No significant differences were found in the infant age and birth weight between diet groups. The characteristics of the study cohorts are summarized in Table 1.

**Table 1:** Characteristics of the study participants.

**Characteristics**	**Overall, n = 70**^**1**^		**Breastfed, n = 30**^**1**^	**Expressed milk-fed, n = 10**^**1**^	**Formula-fed, n = 30**^**1**^	***p*****value**^**2**^
Gender						> 0.9
Female	38 (54 %)		17 (57 %)	5 (50 %)	16 (53 %)	
Male	32 (46 %)		13 (43 %)	5 (50 %)	14 (47 %)	
Mode of delivery						0.015
Caesarean delivery	20 (29 %)		4 (13 %)	2 (20 %)	14 (47 %)	
Vaginal delivery	50 (71 %)		26 (87 %)	8 (80 %)	16 (53 %)	
Age (days)	39 (36, 42)		39 (37, 42)	38 (36, 42)	39 (36, 42)	0.6
Birth weight (g)	3140 (2906, 3310)		3210 (2975, 3460)	3117 (2725, 3233)	3075 (2913, 3255)	0.4
^1^n (%); median (IQR)						
^2^Fisher’s exact test; Kruskal-Wallis test						

### 3.2 Metagenomic characterization of fecal mycobiota

**Fig. 1:**
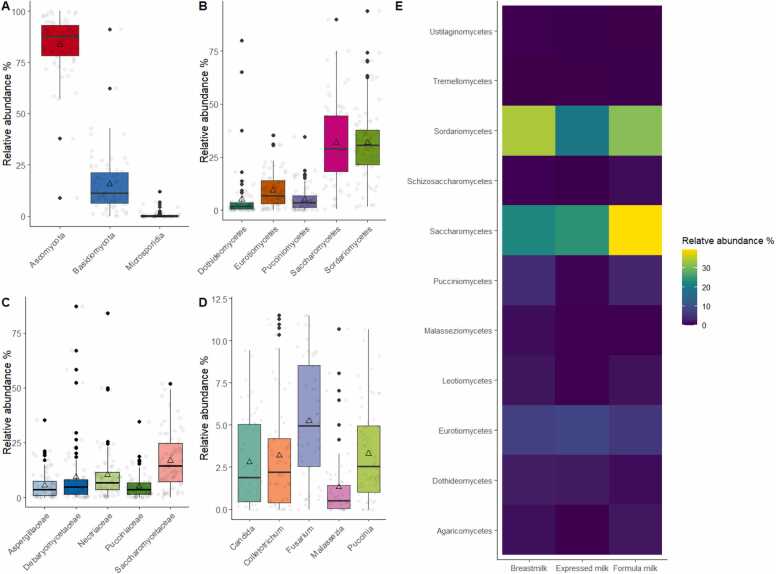
Compositional distribution of fungal features in Hong Kong infants. The figure shows the relative abundance of the top five fungal features at the (A) phylum, (B) class, (C) family, and (D) genus levels. The data is presented in the form of boxplots, with the boxes indicating the 25th to 75th percentile, the line within the box representing the median, the triangle representing the arithmetic mean, and the error bars representing 1.5 × interquartile range. Each dot represents the actual data of the subject. (E) The relative abundance of all fungal classes was plotted as a heatmap based on the group average. The colors of the heatmap represent the relative abundance of each fungal class, with yellow color indicating a higher relative abundance and deep violet color indicating a lower relative abundance.

Illumina sequencing generated a mean of 78,235,336 ± 6728,622 sequencing reads per sample, with 94 % of the reads having a high-quality score greater than Q30. After filtering out low-quality reads, taxonomic profiling revealed that 0.06 % of the reads corresponded to fungal taxonomy, with no significant difference observed between feeding groups (Kruskal-Wallis test, *p* = 0.25; Supplementary Table 1). The overall microbial reads across samples ranged from 13,299,615 to 48,065,789. The number of microbial reads did not differ significantly across feeding groups (Kruskal-Wallis test, *p* = 0.52; Supplementary Table 1). Median microbial reads were 36,453,732 for breastfed samples, 37,948,508 for expressed milk samples, and 36,553,571 for formula-fed samples. Analysis of the gut fungal communities in Hong Kong infants revealed the presence of three dominant fungal phyla: *Ascomycota*, *Basidiomycota*, and *Microsporidia* (Fig. 1A). Among these, *Ascomycota* exhibited the highest average relative abundance (83.7 %), followed by *Basidiomycota* (15.6 %). *Microsporidia* accounted for a lower average relative abundance (0.7 %; Fig. 1A). Further classification revealed the presence of 11 fungal classes, 17 orders, 31 families, 52 genera, and 93 species within the detectable fungal communities.

**Fig. 2:**
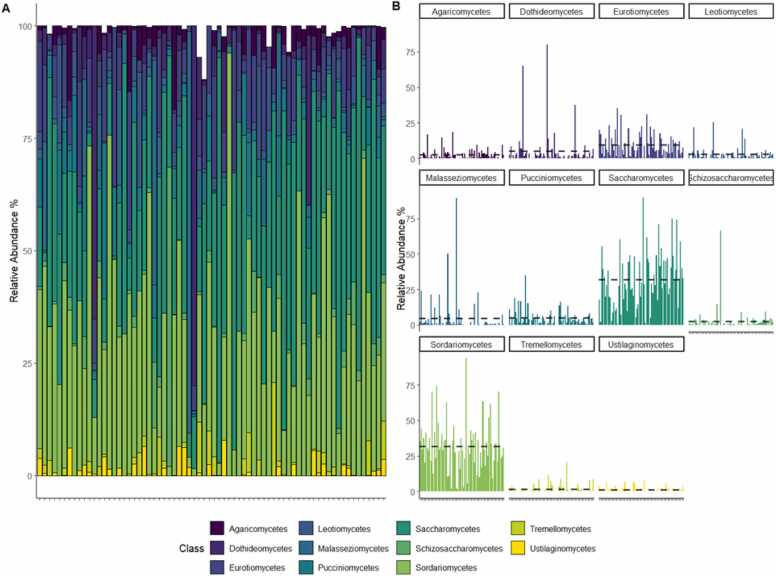
Relative abundance of fungal classes observed in Hong Kong infants. (A) Stacked bar plots display the relative abundances of fungal classes for individual samples. (B). Bar plots display the relative abundances of features stratified by fungal classes for individual samples. Each bar represents a different sample, and the height of the bars corresponds to the relative abundance of the fungal classes. The average relative abundance of each fungal class is represented by a horizontal dashed line.

Among the identified fungal classes, approximately half of the classes, including *Dothideomycetes*, *Eurotiomycetes*, *Leotiomycetes*, *Saccharomycetes*, *Schizosaccharomycetes*, and *Sordariomycetes*, were classified under the phylum *Ascomycota*. The other remaining classes, including *Agaricomycetes, Malasseziomycetes, Pucciniomycetes, Tremellomycetes*, and *Ustilaginomycetes*, were classified under the phylum *Basidiomycota*. The two most abundant classes were *Saccharomycetes* and *Sordariomycetes*, both of which had a relative abundance of 31.8 % (Fig. 1B, Fig. 2A and B). Following these two classes, *Eurotiomycetes* had an average relative abundance of 9.3 %, and *Dothideomycetes* and *Pucciniomycetes* had relative abundances of 5.2 % and 5.0 %, respectively (Fig. 1B, Figs. 2A and 2B). Other detectable fungal classes with less than 5 % relative abundance included *Malasseziomycetes, Leotiomycetes, Agaricomycetes, Schizosaccharomycetes, Tremellomycetes*, and *Ustilaginomycetes* (Figs. 2 A and 2B).

At the taxonomic levels of family and genus, the top five abundant families were *Saccharomycetaceae* (16.9 %), *Nectriaceae* (10.4 %), *Debaryomycetaceae* (9.5 %), *Aspergillaceae* (5.5 %), and *Pucciniaceae* (5.0 %) (Fig. 1C). At the genus level, the top five abundant genera were *Fusarium* (10.4 %), *Candida* (8.4 %), *Colletotrichum* (5.0 %), *Puccinia* (5.0 %), and *Malassezia* (4.8 %) (Fig. 1D). Furthermore, a heatmap was generated to visually represent the overall detected fungal genera and their relative abundance. This heatmap revealed that certain genera exhibited higher abundances in the formula-fed group of infants compared to those of other groups (Fig. 1E).

### 3.3 Diet affects the gut mycobiome diversity in Hong Kong infants

**Fig. 3:**
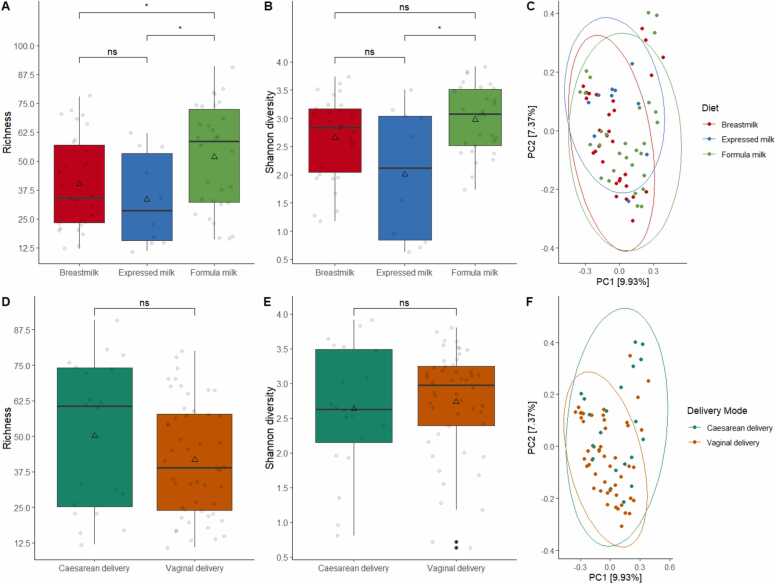
Diversity analyses of the gut mycobiome in Hong Kong infants. (A) Box plots display the observed fungal richness between diet groups. (B) Shannon diversity between diet groups. (C) Principal coordinate analysis (PCoA) plot of Bray-Curtis dissimilarity between diet groups. (D) Box plots illustrates the observed fungal richness between modes of delivery. (E) Shannon diversity between modes of delivery. (F) PCoA plot of Bray Curtis dissimilarity between modes of delivery. Boxes in all plots represent the 25th to 75th percentile, the line within the box represents the median, the triangle represents the arithmetic mean, and the error bars represent 1.5 × interquartile range. Each dot represents the actual data of the subject. *p* values were calculated based on the Mann-Whitney *U* test. ns: not significant, * : *p* < 0.05.

We compared the fungal diversities of the gut mycobiomes in Hong Kong infants and compared them based on diet groups and delivery modes. Alpha and beta diversities were used to analyze differences between the breastfed, expressed milk-fed, and formula-fed groups (Figs. 3A-C). Alpha diversity analysis showed that formula-fed infants had the highest diversity, with greater richness and Shannon diversity median values compared with those of the other two diet groups ([Richness] median = 58.5; [Shannon diversity] median = 3.1; Fig. 3A-B). The expressed milk group showed a significant decrease in both richness and Shannon diversity ([Richness] median = 28.5; expressed milk Shannon diversity median = 2.1; *p* = 0.02; Fig. 3A-B). The breastfed group only showed a difference in richness when compared to the formula-fed group (breastfed vs formula-fed = 34 vs 58.5; *p* = 0.04; Fig. 3A), with no significant difference in diversity (Fig. 3B). Beta diversity analysis also revealed significant compositional differences between the diet groups (PERMANOVA *p* = 0.04), and pairwise PERMANOVA indicated a significant difference between the formula milk and expressed milk groups (*p* = 0.05). No significant differences were observed between the breastfed and expressed milk-fed groups (*p* = 0.19), or between the formula-fed and breastfed groups (*p* = 0.09). The PCoA plot showed a distinct separation of the expressed milk group from the other diet groups (Fig. 3C).

To account for potential confounding effects, we analyzed the impact of delivery mode on gut mycobiome diversity and performed multiple linear regressions to examine the association between diversities and diet groups. Univariate analyses did not reveal a significant difference in alpha diversity between the C-section and vaginal delivery infants ([Richness] *p* = 0.20; [Shannon diversity] *p* = 0.65; Fig. 3D-E; Table 2). However, a compositional difference was found between the two groups (Bray-Curtis dissimilarity, PERMANOVA *p* = 0.01; Fig. 3F). Therefore, we adjusted the analysis for potential confounders, including mode of delivery, sex, and birth weight. After adjustment, the association between alpha diversities and diet groups remained significant in the multivariable linear regression model (*p* < 0.05, Table 2). The expressed milk group showed significantly lower Shannon diversity, even after accounting for potential confounders (adjusted beta coefficient = −0.67, 95 % CI = −1.2 to −0.12, *p* = 0.02, Table 2). The formula milk group also showed a marginal significance in Shannon diversity, with higher diversity observed (adjusted beta coefficient = 0.4, 95 % CI = −0.01–0.82, *p* = 0.06, Table 2). Similarly, a marginal significance was found in richness measurement, with the formula-fed group showing higher richness compared with that in the breastfed group (adjusted beta coefficient = 11.0, 95 % CI = −0.53–23, *p* = 0.06; Table 2). We also assessed the interaction between infant feeding diets and delivery mode, but no significant interaction effect was found in the affecting alpha diversities (*p* = 0.2). Compositional differences were further analyzed using multivariable PERMANOVA, which confirmed that the effect of diet on fungal composition remained significant even after adjusting for potential confounders (R^2^ = 0.036, *p* = 0.04; Table 3). No interaction effect was found between the delivery mode and diet on fungal beta diversity (*p* = 0.42).

**Table 2:** Multivariable linear regression analyses of potential clinical factors associated with gut mycobiome richness and Shannon diversity.

**Characteristics**	**Shannon's diversity**							**Richness**					
	**Univariate**			**Multivariable**				**Univariate**			**Multivariable**		
	**beta**	**95 % CI**^a^	***p*****value**	**beta**	**95 % CI**^a^	***p*****value**		**beta**	**95 % CI**^a^	***p*****value**	**beta**	**95 % CI**^a^	***p*****value**
Diet													
Breastfed	—	—		—	—			—	—		—	—	
Expressed milk-fed	−0.66	−1.2, −0.11	0.019	−0.67	−1.2, −0.12	0.019		−6.7	−22, 8.8	0.4	−5.7	−22, 10	0.5
Formula-fed	0.32	−0.07, 0.70	0.11	0.4	−0.01, 0.82	0.056		12	0.83, 23	0.035	11	−0.53, 23	0.061
Mode of delivery													
Caesarean delivery	—	—		—	—			—	—		—	—	
Vaginal delivery	0.1	−0.33, 0.53	0.6	0.31	−0.11, 0.73	0.15		−8.4	−20, 3.1	0.2	−3.3	−16, 8.8	0.6
Gender													
Female	—	—		—	—			—	—		—	—	
Male	0.24	−0.15, 0.62	0.02	0.27	−0.09, 0.63	0.14		2.77	−7.8, 13.3	0.6	1.7	−8.8, 12	0.8
Birth weight (g)	0	0.00, 0.00	0.98	0	0.00, 0.00	0.8		0.01	−0.01, 0.02	0.3	0.01	−0.01, 0.02	0.3

a CI, confidence interval

**Table 3:** Permutational multivariate analysis of variance (PERMANOVA) table for gut mycobiome of Hong Kong infants based on Bray-Curtis dissimilarity.

**Characteristics**	**Df**	***Pseudo-F*****value**	**R**^**2**^	***p*****value**
Diet	2	1.270	0.036	0.044
Mode of delivery	1	1.423	0.020	0.033
Gender	1	1.274	0.018	0.085
Birth weight (g)	1	1.059	0.015	0.325
Residuals	64		0.910	
Total	69			

Df, degrees of freedom; R^2^, R squared

### 3.4 Diet affects the gut mycobiome compositions in Hong Kong infants

**Fig. 4:**
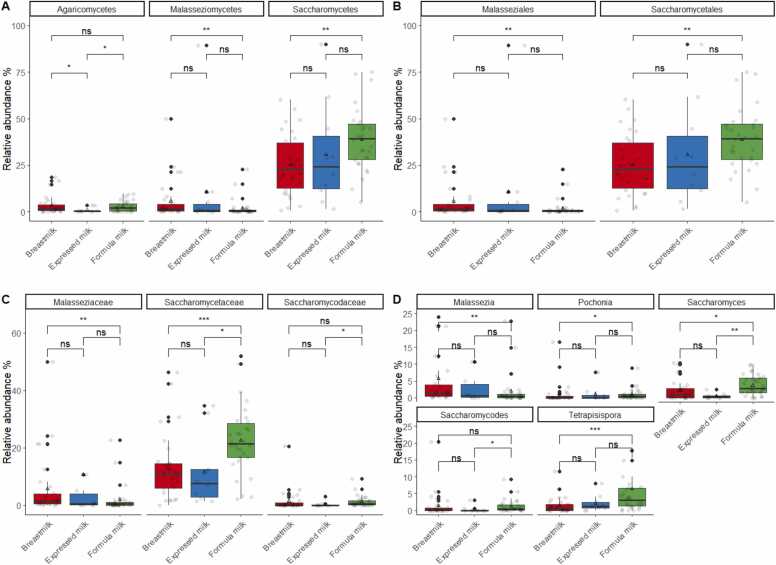
Relative abundance analysis of diet-associated gut mycobiome features. Comparisons of the relative abundance of diet-associated fungal features (Kruskal Wallis test *p* < 0.05) at the level of (A) class, (B) order, (C) family, and (D) genus between breastfed, expressed milk-fed, and formula-fed infants. Boxes in all plots represent the 25th to 75th percentile, the line within the box represents the median, the triangle represents the arithmetic mean, and the error bars represent 1.5 × interquartile range. Each dot represents the actual data of the subject. *p* values were calculated using the Mann-Whitney *U* test. ns: not significant, * : *p* < 0.05, * *: *p* < 0.01, * ** : *p* < 0.001.

Differences in the relative abundance of fungal taxa between different diet groups were compared using the Kruskal-Wallis and post-hoc Dunn’s tests. At the phylum level, there was no significant difference observed for *Ascomycota* (*p* = 0.28), *Basidiomycota* (*p* = 0.16), and *Microsporidia* (*p* = 0.49). However, at the class level, *Agaricomycetes*, *Malasseziomycetes*, and *Saccharomycetes* were found to be significantly different (Kruskal-Wallis test *p* < 0.05, Table 4, Fig. 4A). The median relative abundance of *Agaricomycetes* was lower in the expressed milk-fed group compared to that in the breastfed group (breastmilk = 1.45 %, expressed milk = 0.36 %, FDR corrected *p* = 0.04, Table 4, Fig. 4A). In the formula-fed group, *Malasseziomycetes* was significantly lower than that in the breastfed group (breastmilk = 1.4 %, formula milk = 0.43 %; FDR corrected *p* = 0.01, Table 4, Fig. 4A), but enrichment of relative abundance was observed for *Saccharomycetes* (breastmilk = 22.6 %, formula milk = 39.3 %, FDR corrected *p* = 0.01, Table 4, Fig. 4A). Likewise, the orders of *Malasseziomycetes* and *Saccharomycetes*, namely *Malasseziales* and *Saccharomycetales*, exhibited significant differences between diet groups (FDR corrected *p* = 0.01, Table 4, Fig. 4B). Similarly, family-level analyses using the Kruskal-Wallis test indicated significant differences among *Malasseziaceae* (*p* = 0.01), *Saccharomycetaceae* (*p* = 0.001), and *Saccharomycodaceae* (*p* = 0.03) (Table 4, Fig. 4C). At the genus level, the Kruskal-Wallis test revealed significant differences between diet groups for *Malassezia* (*p* = 0.01), *Pochonia* (*p* = 0.05), *Saccharomyces* (*p* = 0.002), *Saccharomycodes* (*p* = 0.03), and *Tetrapisispora* (*p* = 0.002) (Table 4, Fig. 4D). Among these, *Pochonia* (FDR corrected *p* = 0.05), *Saccharomyces* (FDR corrected *p* = 0.03), and *Tetrapisispora* (FDR corrected *p* = 0.002) were significantly higher in formula-fed infants (Table 4), whereas the relative abundance of *Malassezia* was significantly lower (FDR corrected *p* = 0.01, Table 4).

**Table 4:** Fungal features significantly associated with diet groups.

**Fungal taxonomy**	**Breastfed**		**Expressed milk-fed**			**Formula-fed**				
	**Median**	**IQR**	**Median**	**IQR**	**FDR corrected*****p***	**Median**	**IQR**	**FDR corrected*****p***		***p*****value (Kruskal-Wallis)**
Class										
*Agaricomycetes*	1.45	(0.51–3.84)	0.36	(0–0.51)	0.040	2.08	(0.42–4.35)	0.824		0.048
*Malasseziomycetes*	1.42	(0.51–3.92)	0.56	(0.07–3.97)	0.250	0.43	(0–1.07)	0.010		0.013
*Saccharomycetes*	22.61	(12.59–36.94)	24.26	(12.27–40.74)	0.651	39.34	(28.16–47.01)	0.009		0.010
Order										
*Malasseziales*	1.42	(0.51–3.92)	0.56	(0.07–3.97)	0.250	0.43	(0–1.07)	0.010		0.013
*Saccharomycetales*	22.61	(12.59–36.94)	24.26	(12.27–40.74)	0.651	39.34	(28.16–47.01)	0.009		0.010
Family										
*Malasseziaceae*	1.42	(0.51–3.92)	0.56	(0.07–3.97)	0.250	0.43	(0–1.07)	0.010		0.013
*Saccharomycetaceae*	10.86	(5.91–14.47)	7.6	(2.91–12.43)	0.690	21.4	(16.72–28.53)	0.002		0.001
*Saccharomycodaceae*	0.04	(0–0.79)	0	(0–0.07)	0.221	0.47	(0.09–1.62)	0.098		0.025
Genus										
*Malassezia*	1.42	(0.51–3.92)	0.56	(0.07–3.97)	0.250	0.43	(0–1.07)	0.010		0.013
*Pochonia*	0	(0–0.38)	0.04	(0–0.71)	0.599	0.5	(0.03–1.08)	0.045		0.048
*Saccharomyces*	0.95	(0–2.72)	0.21	(0–0.57)	0.102	2.69	(1.49–5.78)	0.032		0.002
*Saccharomycodes*	0.04	(0–0.79)	0	(0–0.07)	0.221	0.47	(0.09–1.62)	0.098		0.025
*Tetrapisispora*	0.6	(0–1.84)	1.19	(0.74–2.39)	0.336	2.99	(1.27–6.62)	0.002		0.002

FDR-P, false discovery rate adjusted P value; IQR, interquartile range

The Kruskal-Wallis test was used to examine the relative abundance of fungal features across diet groups in Hong Kong infants.

The *post hoc* Dunn’s test was conducted to identify significant differences relative to the reference breastmilk group.

**Fig. 5:**
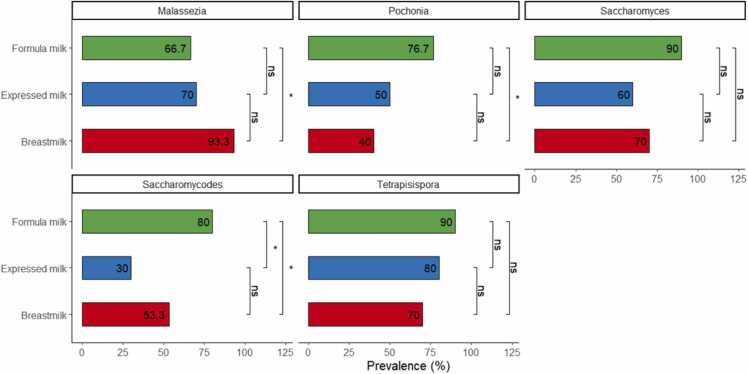
Prevalence comparisons of diet-associated fungal genera. Bar plots indicate the percentage of each diet-associated genus present in each diet group. *p* values were calculated based on the pairwise chi-square test. ns: not significant, * : *p* < 0.05.

The fungal genera showing significant differences in relative abundance were further subjected to multivariable logistic regressions and prevalence analysis (Table 5, Fig. 5). Logistic regression models showed that the diet-associated genera *Malassezia*, *Pochonia*, and *Saccharomyces* remained significant even after adjustments (Table 5, *p* < 0.05). Consistent with the observations on relative abundance, the genus *Malassezia* was found to be significantly lower in the formula-fed group compared to that in the direct breastfed group (breastmilk vs. formula milk prevalence = 93.3 % vs. 70 %, aORs = 0.13, 95 % CI=0.02–0.59, *p* = 0.02, Fig. 5, Table 5). In contrast, *Pochonia* and *Saccharomyces* were significantly higher in formula-fed infants ([*Pochonia*] breastmilk vs. formula milk prevalence = 40 % vs. 76.7 %, aORs = 6.47, 95 % CI=1.90–25.5, *p* = 0.004; [*Saccharomyces*] breastmilk vs. formula milk prevalence = 70 % vs. 90 %, aORs = 5.34, 95 % CI = 1.20–32.1, *p* = 0.04, Table 5, Fig. 5). *Tetrapisispora* showed marginally significant differences in the formula-fed group compared to that in the direct breastfed group (breastmilk vs formula milk prevalence = 70 % vs. 90 %, aORs = 5.16, 95 % CI = 1.09–32.5, *p* = 0.053, Table 5, Fig. 5).

**Table 5:** Multivariable logistic regression models testing fungal genera associated with diet adjusted for covariates.

**Characteristics**	***Malassezia***			***Pochonia***			***Saccharomyces***			***Tetrapisispora***		
	aORs	95 % CI	*p* value	aORs	95 % CI	*p* value	aORs	95 % CI	*p* value	aORs	95 % CI	*p* value
Diet												
Breastfed	—	—		—	—		—	—		—	—	
Expressed milk	0.15	0.02, 1.12	0.066	1.47	0.31, 7.08	0.6	0.64	0.14, 3.22	0.6	2.72	0.44, 26.1	0.3
Formula	0.13	0.02, 0.59	0.017	6.47	1.90, 25.5	0.004	5.34	1.20, 32.1	0.041	5.16	1.09, 32.5	0.053
Mode of Birth												
C-section	—	—		—	—		—	—		—	—	
Vaginal delivery	0.77	0.19, 2.88	0.7	1.83	0.52, 7.06	0.4	2.38	0.56, 11.0	0.2	0.56	0.07, 2.92	0.5
Gender												
Female	—	—		—	—		—	—		—	—	
Male	1.52	0.45, 5.48	0.5	2.77	0.96, 8.58	0.066	1.19	0.35, 4.15	0.8	1.28	0.34, 5.15	0.7
Birth weight (g)	1	1.00, 1.00	0.8	1	1.00, 1.00	> 0.9	1	1.00, 1.00	0.8	1	1.00, 1.00	0.02

aOR, adjusted Odds Ratio; CI, confidence interval

## 4 Discussion

This study is the first to demonstrate that the infant feeding diet has a substantial impact on the gut mycobiome during early life. Although the influence of diet on the gut bacteriome in infancy has been extensively studied [24], [25], [26], [27], [50], its effects on the gut mycobiome have remained largely unexplored. Previous studies have investigated various factors associated with the infant mycobiome, including body mass index, probiotics, atopic dermatitis, antibiotics, asthma, type 1 diabetes, and environmental influence [28], [61], [62], [63], [64], [65], [66]. However, only one study has examined the influence of postnatal diet on the gut mycobiome using piglets as an animal model [67], indicating a significant gap in our understanding of the associations between diet and the gut mycobiome in early life [68], [69]. To address this knowledge gap, the current study focused on infants who were fed through direct breastfeeding, expressed milk feeding, or formula feeding. Our findings revealed that formula feeding was associated with increased diversity and enrichment of fungal components in the gut mycobiome (Fig. 3, Fig. 4, Fig. 5), consistent with earlier observations from studies on the gut bacteriome [70], [71], [72], [73]. In contrast, the diversity and composition between the breastfed and expressed milk-fed groups was not substantially different (Fig. 3, Fig. 4, Fig. 5). These findings indicate that the feeding type plays a crucial role in shaping the gut mycobiome of infants.

Formula-fed infants exhibited higher levels of gut mycobiome diversity, as indicated by higher alpha diversities compared to those in the breastfed and expressed milk-fed infants. Specifically, formula-fed infants displayed significantly higher levels of richness and Shannon diversity compared with those in the other two feeding groups (Fig. 3A-B). In contrast, breastfed infants and those fed expressed milk did not differ significantly in terms of mycobiome diversity (Fig. 3A-B). Further analyses, accounting for potential confounding effects, revealed that formula feeding was associated with higher levels of richness, diversities, and different relative abundances in fungal communities (Fig. 3 A-B, Fig. 4, Table 2, and Table 4). These findings further support that formula milk is linked with enrichment of the fungal community in the gut, likely because of the higher nutritional content in formula milk compared to that in breastmilk [70], [71], [72], [74]. This increase in the gut mycobiome is similar to that observed in the gut microbiome of formula-fed infants, where higher microbial diversity has been attributed to the greater protein levels in formula milk and the absence of human milk oligosaccharides (HMOs) [15], [75], [76], [77]. Infant formulas may contain supplements such as short-chain galactooligosaccharides and long-chain fructooligosaccharides. However, these supplements are not as selective as HMOs, suggesting that the nutritional specificity of breastmilk, including the presence of HMOs, may play a role in shaping the gut bacteriome and mycobiome of infants [24], [78], [79]. In contrast, the fungal community of the expressed milk feeding group was generally similar to that of the breastfeeding group, with no remarkable differences observed (Fig. 3A-B and Table 2). This similarity is likely because of the identical nutritional composition between these two groups, resulting in similar levels of diversity between breastfed and expressed milk-fed infants.

Distinct differences in fungal genera were observed across feeding modes. However, limited studies have examined the impact of diet on the infant gut mycobiome, hindering direct comparison with our findings. Nonetheless, previous research on the human breastmilk mycobiome has provided insights into the gut mycobiome in early infancy [34], [68], [69]. For example, Boix-Amorós et al. investigated breastmilk samples from different countries and showed that *Malassezia* was significantly less abundant in South African samples, whereas Chinese milk samples exhibited a higher relative abundance of *Malassezia*. These findings suggest that geographical, dietary, and cultural factors may contribute to differences in the fungal composition of breastmilk [34]. Similar to these findings, our study showed a higher relative abundance of *Malassezia* in Hong Kong infants who were exclusively breastfed (Fig. 4D and Table 4). As *Malassezia* is a major component of the fungal skin flora, colonization of the infant gut by *Malassezia* may be attributed to the transfer of fungi from the skin of the mother's breast to the infant gut [80]. Further, we found that formula-fed infants exhibited significant increases in the relative abundance and prevalence of *Saccharomyces*, *Pochonia*, and *Tetrapisispora* (Table 4; Table 5; Fig. 4 D; Fig. 5). These fungal genera may have been enriched owing to differences in the nutritional compositions of formula milk, such as animal-based or soy-based formulas, the inclusion of prebiotics and/or probiotics, or other supplemental ingredients leading to distinct fungal profiles [81], [82]. The use of probiotics is gaining increasing popularity in infant formulas and/or food to support healthy growth and development [83]. For example, the probiotic strain *Saccharomyces boulardii* has demonstrated efficacy in preventing and treating various gastrointestinal diseases in both adults and children [84]. Previous studies indicated that *S. boulardii*-supplemented formula was well-tolerated by preterm infants and conferred beneficial effects on the gut microbiome, promoted accelerated weight gain, and improved feeding tolerance [85], [86]. Nevertheless, further research, including double-blinded placebo-controlled trials, is essential to verify the safety and long-term benefits of probiotic-enriched formulas for infants. Notably, *Pochonia* has been previously reported in the gut mycobiome of humans [27]. Soybean, a crop where *Pochonia* is used as a biological control agent to promote growth [87], may contribute to the elevated levels of *Pochonia* found in formula-fed infants, as some infant formulas contain soybean components [88], [89]. No notable differences in fungal composition were observed between the breastfeeding and expressed milk feeding groups (Fig. 4D and Table 4). Currently, limited information is available on the mycobiome of expressed milk in the literature, and its impact on infant health remains poorly understood. A recent study identified expressed breastmilk as a potential risk factor for neonatal yeast colonization in intensive care settings, suggesting that the consumption of expressed breastmilk may contribute to the acquisition of *Candida*, which is associated with invasive fungal infections [90]. However, in our study, analysis of the gut mycobiome showed no statistical significance in the relative abundance and prevalence of *Candida* species between the expressed milk group and two other feeding groups among the Hong Kong Chinese infants (Supplementary Figure 1), suggesting that *Candida* colonization during infancy may not be solely limited to transmission through expressed milk. However, further studies are necessary to explore the potential health implications of these findings.

Our study has several limitations. The small sample size in the expressed milk group reduces statistical power, indicating the need for larger samples to confirm the findings. Further, using only one curated fungal database to estimate fungal abundance may not fully capture fungal communities. Experimental verification, such as fungal culture and quantitative PCR, can enhance the robustness of these findings. Additional studies with increased participant numbers, comprehensive data on the family environment, and detailed analysis of infant formula ingredients are essential for a thorough understanding of the gut mycobiome in early life and its dietary interactions.

## 5 Conclusion

Our analysis revealed, for the first time, that formula-fed infants had higher fungal diversity and composition in their gut mycobiome compared to those of breastfed and expressed milk-fed infants. However, no significant differences were observed between the breastfed and expressed milk-fed groups. Given the small sample size in expressed milk group, the findings of this group should be considered preliminary. Despite this limitation, the study provides additional evidence of the correlation between feeding methods and gut mycobiome changes, emphasizing the significance of the gut mycobiome during infancy, and of strengthening maternal awareness to choose appropriate feeding methods, aligning with the increasing promotion and encouragement of breastfeeding practice among healthcare professionals and the general public, owing to its physiological benefits.

## Ethics approval statement

Written informed consent was obtained from all participants in accordance with the Declaration of Helsinki guidelines. The study protocols were approved by the University of Hong Kong and the Hospital Authority Hong Kong West Cluster (UW 18–035 & UW 21–752).

## Author statement

Each named author has substantially contributed to conducting the underlying research and drafting this manuscript. Additionally, the named authors have declared no conflict of interest, financial or otherwise. All authors approved the submission to CSBJ.

## CRediT authorship contribution statement

**Teng Jade Lee Lee:** Writing – original draft, Supervision, Investigation, Funding acquisition, Conceptualization. **Fong Jordan Yik Hei:** Writing – original draft, Methodology, Investigation, Formal analysis. **Lok Kris Yuet Wan:** Writing – review & editing, Methodology, Funding acquisition, Conceptualization. **Woo Patrick Chiu Yat:** Writing – review & editing. **Yeung Man Lung:** Writing – review & editing, Supervision. **Li Wing Ho:** Writing – review & editing, Formal analysis.

## Declaration of Competing interest

The authors declare no competing interests.

## Data Availability

Shotgun sequencing-generated fastq data were deposited into the Sequence Read Archive (SRA) of NCBI under BioProject accession number PRJNA1129277 and will be accessible once they become publicly available upon publication.
