# Supplementary material for: Impact of diet on the gut mycobiome of Hong Kong Chinese infants
Source: Comput Struct Biotechnol J. 2025 Feb 14;27:661–71. doi: 10.1016/j.csbj.2025.02.006 (PMC11889518; doi:10.1016/j.csbj.2025.02.006)
Supplement: Supplementary file 1 — Supplementary material [file mmc1.docx]

Supplementary Table 1. Microbial reads and fungal proportion across sample groups.

| **Sample** | **Group** | **Microbial reads** | **Fungal reads** | **Fungal reads proportion** |
| --- | --- | --- | --- | --- |
| T1002 | Breastfed | 38710829 | 574 | 1.48E-05 |
| T1005 | Breastfed | 34863680 | 507 | 1.45E-05 |
| T1007 | Breastfed | 30635265 | 8002 | 0.0002612 |
| T1014 | Breastfed | 34183703 | 175 | 5.12E-06 |
| T1023 | Breastfed | 35212999 | 195 | 5.54E-06 |
| T1024 | Breastfed | 32677929 | 737 | 2.26E-05 |
| T1025 | Breastfed | 39812477 | 609 | 1.53E-05 |
| T1026 | Breastfed | 40538602 | 204 | 5.03E-06 |
| T1028 | Breastfed | 29476261 | 3832 | 0.00013 |
| T1033 | Breastfed | 35578009 | 51 | 1.43E-06 |
| T1036 | Breastfed | 37365552 | 419 | 1.12E-05 |
| T1042 | Breastfed | 34575074 | 228 | 6.59E-06 |
| T1045 | Breastfed | 41399370 | 196 | 4.73E-06 |
| T1051 | Breastfed | 41701867 | 504 | 1.21E-05 |
| T1052 | Breastfed | 34224983 | 70 | 2.05E-06 |
| T1057 | Breastfed | 37540141 | 374 | 9.96E-06 |
| T1058 | Breastfed | 35261937 | 415 | 1.18E-05 |
| T1068 | Breastfed | 43680916 | 110 | 2.52E-06 |
| T1070 | Breastfed | 44926371 | 230 | 5.12E-06 |
| T1071 | Breastfed | 29212035 | 5548 | 0.0001899 |
| T1074 | Breastfed | 35371615 | 39 | 1.10E-06 |
| T1076 | Breastfed | 42701633 | 1227 | 2.87E-05 |
| T1079 | Breastfed | 37329455 | 300 | 8.04E-06 |
| T1080 | Breastfed | 33564885 | 262 | 7.81E-06 |
| T1082 | Breastfed | 42699524 | 226 | 5.29E-06 |
| T1083 | Breastfed | 37850241 | 62 | 1.64E-06 |
| T1088 | Breastfed | 38244085 | 392 | 1.02E-05 |
| T1089 | Breastfed | 27520239 | 7358 | 0.0002674 |
| T1095 | Breastfed | 38903605 | 762 | 1.96E-05 |
| T1098 | Breastfed | 32965595 | 424 | 1.29E-05 |
| T2010 | Expressed milk | 41976303 | 169 | 4.03E-06 |
| T2012 | Expressed milk | 40784065 | 557 | 1.37E-05 |
| T2016 | Expressed milk | 33838622 | 169 | 4.99E-06 |
| T2027 | Expressed milk | 33183341 | 2252 | 6.79E-05 |
| T2036 | Expressed milk | 44153861 | 41 | 9.29E-07 |
| T2043 | Expressed milk | 38028223 | 1333 | 3.51E-05 |
| T2049 | Expressed milk | 37868792 | 5312 | 0.0001403 |
| T2051 | Expressed milk | 35434010 | 675 | 1.90E-05 |
| T2053 | Expressed milk | 40268449 | 266 | 6.61E-06 |
| T2054 | Expressed milk | 37335386 | 196 | 5.25E-06 |
| T3004 | Formula milk | 48065789 | 29 | 6.03E-07 |
| T3005 | Formula milk | 36358062 | 1341 | 3.69E-05 |
| T3016 | Formula milk | 42562206 | 163 | 3.83E-06 |
| T3017 | Formula milk | 37413938 | 123 | 3.29E-06 |
| T3025 | Formula milk | 41750217 | 736 | 1.76E-05 |
| T3026 | Formula milk | 43124941 | 55 | 1.28E-06 |
| T3027 | Formula milk | 40581206 | 50 | 1.23E-06 |
| T3031 | Formula milk | 41623577 | 53 | 1.27E-06 |
| T3039 | Formula milk | 35975971 | 256 | 7.12E-06 |
| T3043 | Formula milk | 33057696 | 1914 | 5.79E-05 |
| T3044 | Formula milk | 31591387 | 627 | 1.98E-05 |
| T3051 | Formula milk | 23850809 | 8064 | 0.0003381 |
| T3053 | Formula milk | 33067015 | 265 | 8.01E-06 |
| T3056 | Formula milk | 38650483 | 259 | 6.70E-06 |
| T3059 | Formula milk | 40375301 | 351 | 8.69E-06 |
| T3062 | Formula milk | 39081947 | 1194 | 3.06E-05 |
| T3065 | Formula milk | 13299615 | 13463 | 0.0010123 |
| T3066 | Formula milk | 36269814 | 1155 | 3.18E-05 |
| T3071 | Formula milk | 34973837 | 770 | 2.20E-05 |
| T3072 | Formula milk | 16385834 | 13132 | 0.0008014 |
| T3073 | Formula milk | 35170486 | 777 | 2.21E-05 |
| T3076 | Formula milk | 40099170 | 829 | 2.07E-05 |
| T3077 | Formula milk | 31316749 | 1120 | 3.58E-05 |
| T3080 | Formula milk | 36749079 | 1347 | 3.67E-05 |
| T3083 | Formula milk | 37859797 | 530 | 1.40E-05 |
| T3086 | Formula milk | 39709599 | 217 | 5.46E-06 |
| T3087 | Formula milk | 31367928 | 2455 | 7.83E-05 |
| T3088 | Formula milk | 34546019 | 2245 | 6.50E-05 |
| T3090 | Formula milk | 39802201 | 2296 | 5.77E-05 |
| T3091 | Formula milk | 33221936 | 873 | 2.63E-05 |
