# Supplementary material for: Impact of diet on the gut mycobiome of Hong Kong Chinese infants
Source: Comput Struct Biotechnol J. 2025 Feb 14;27:661–71. doi: 10.1016/j.csbj.2025.02.006 (PMC11889518; doi:10.1016/j.csbj.2025.02.006)
Supplement: Supplementary file 2 — Supplementary material [file mmc2.docx]

**Supplementary information**


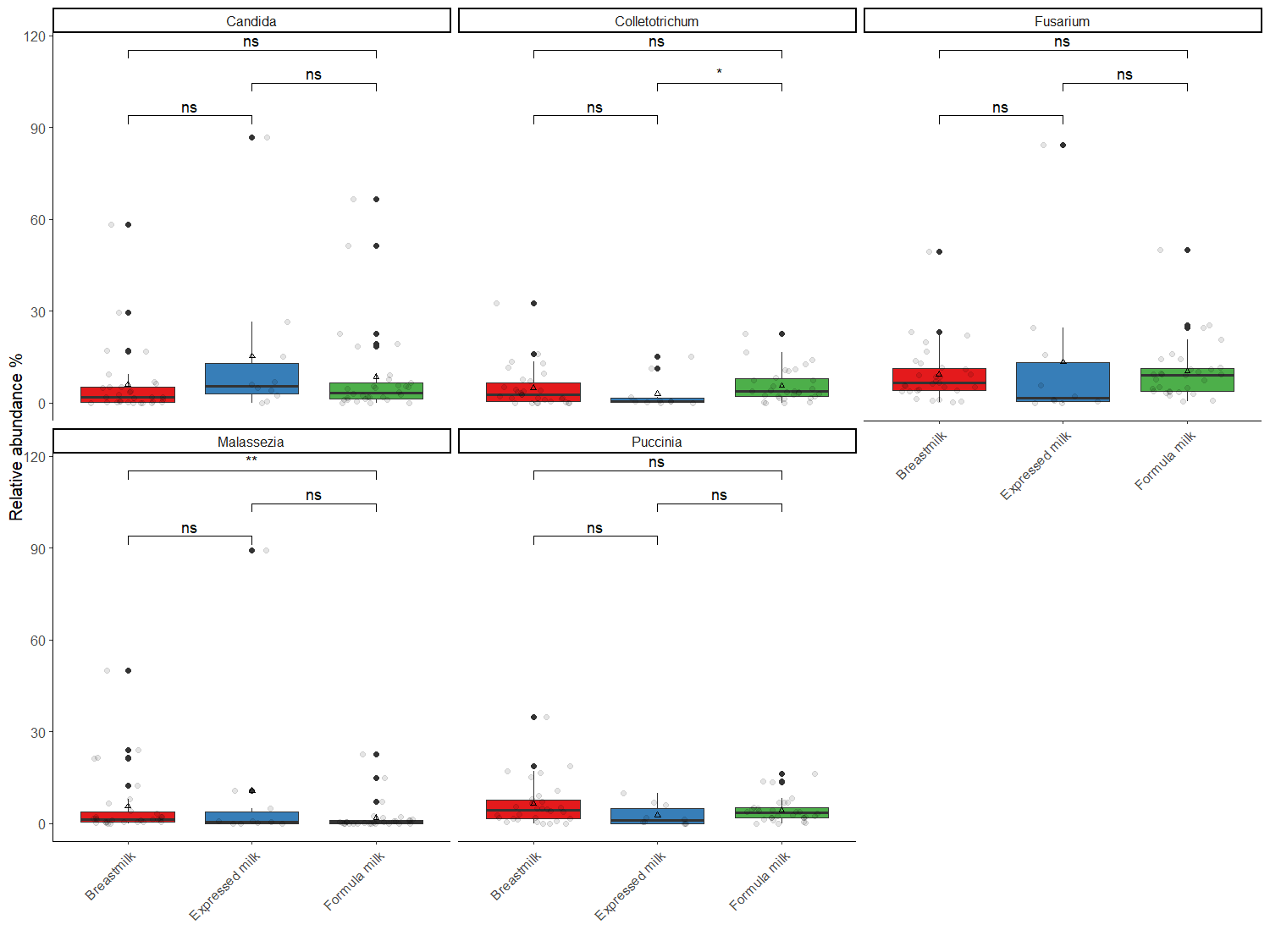


Supplementary Figure 1. Comparison of relative abundance of the top five most abundant gut fungal genera in breastmilk, expressed breastmilk, and formula milk groups. Boxes in all plots represent the 25^th^ to 75^th^ percentile, the line within the box represents the median, and the triangle representing the arithmetic mean. Error bars represents 1.5× interquartile range. Each dot represents the actual data of the subject. *p* values were calculated using the Mann-Whitney U test.
